# A higher number of SARS-COV-2 infections in quilombola communities than in the local population in Brazil

**DOI:** 10.3389/fpubh.2023.1095162

**Published:** 2023-05-26

**Authors:** Aline Fagundes Martins, Daniela Raguer Valadão de Souza, José Melquiades de Rezende Neto, Aryanne Araujo Santos, Grazielly Bispo da Invenção, Igor Leonardo Santos Matos, Kezia Alves dos Santos, Pamela Chaves de Jesus, Francilene Amaral da Silva, Fernando Henrique Oliveira de Almeida, Fernando Yuri Nery do Vale, Dennyson Leandro M. Fonseca, Lena F. Schimke, Saulo Santos Matos, Brenda Morais Oliveira, Cyntia Silva Ferreira, Bruna de Paula Dias, Samara Mayra Soares Alves dos Santos, Camila Cavadas Barbosa, Ikaro Daniel de Carvalho Barreto, Ana Karolina Mendes Moreno, Ricardo Lemes Gonçalves, Breno de Mello Silva, Otavio Cabral-Marques, Lysandro Pinto Borges

**Affiliations:** ^1^Department of Pharmacy, Federal University of Sergipe, São Cristóvão, SE, Brazil; ^2^Department of Clinical and Toxicological Analyses, School of Pharmaceutical Sciences University of São Paulo, São Paulo, Brazil; ^3^Interunit Postgraduate Program on Bioinformatics, Institute of Mathematics and Statistics (IME), University of São Paulo (USP), São Paulo, SP, Brazil; ^4^Department of Immunology, Institute of Biomedical Sciences, University of São Paulo, São Paulo, SP, Brazil; ^5^Department of Biological Sciences, Federal University of Ouro Preto, Ouro Preto, Minas Gerais, Brazil; ^6^UFRPE, Recife, Pernambuco, Brazil; ^7^Postgraduate Program in Animal Biodiversity, Institute of Biological Sciences, Federal University of Goiás, Goiânia, Brazil; ^8^Department of Pharmacy and Postgraduate Program of Health and Science, Federal University of Rio Grande do Norte, Natal, Brazil; ^9^Department of Medicine, Division of Molecular Medicine, University of São Paulo School of Medicine, São Paulo, Brazil; ^10^Laboratory of Medical Investigation 29, University of São Paulo School of Medicine, São Paulo, Brazil

**Keywords:** anti-SARS-CoV-2 antibodies, COVID-19, quilombola, quilombola communities, risk factors

## Abstract

The historical and social vulnerability of quilombola communities in Brazil can make them especially fragile in the face of COVID-19, considering that several individuals have precarious health systems and inadequate access to water. This work aimed to characterize the frequency of SARS-COV-2 infections and the presence of IgM and IgG SARS-CoV-2 antibodies in quilombola populations and their relationship with the presence of risk factors or preexisting chronic diseases in the quilombola communities. We analyzed the sociodemographic and clinical characteristics, serological status, comorbidities, and symptoms of 1,994 individuals (478 males and 1,536 females) from 18 Brazilian municipalities in the State of Sergipe of quilombola communities, which were evaluated at different epidemiological weeks, starting at the 32nd (August 6th) and ending at the 40th (October 3rd) epidemiological week. More than 70% of studied families live in rural areas and they have an extreme poverty social status. Although we found a higher number of SARS-COV-2 infections in quilombola communities than in the local population, their SARS-CoV-2 reactivity and IgM and IgG positivity varied across the communities investigated. Arterial hypertension was the most risk factor, being found in 27.8% of the individuals (9.5% in stage 1, 10.8% in stage 2, and 7.5% in stage 3). The most common COVID-19 symptoms and comorbidities were headache, runny nose, flu, and dyslipidemia. However, most individuals were asymptomatic (79.9%). Our data indicate that mass testing must be incorporated into public policy to improve the health care system available to quilombola populations during a future pandemic or epidemic.

## Introduction

On January 30, 2020, the World Health Organization (WHO) declared a pandemic due to discovering a new type of coronavirus in China (1). So far, more than 650 million individuals have been infected by the severe acute respiratory syndrome virus 2 (SARS-CoV-2), causing more than 6.6 million deaths. Like several countries, Brazil’s health system has been overwhelmed by the pandemic. The country’s economic inequality has also exacerbated the impact of the pandemic on vulnerable populations (2, 3). Regarding total cases and deaths, Brazil has been one of the countries most affected by COVID-19. Until March 2023, Brazil had recorded over 37 million patients and more than 699,000 deaths, making it the second-highest number of COVID-19 deaths worldwide. At the study period, according to data from the Brazilian Ministry of Health, during the period from August 2020 to October 2020, Brazil had a significant increase in the number of COVID-19 cases and deaths as follows: August 2020: 3,669,995 confirmed cases and 118,649 deaths, September 2020: 4,847,092 confirmed cases and 143,962 deaths, October 2020: 5,394,128 confirmed cases and 157,981 deaths (4–7).

Critically, there are several economically disadvantaged communities in Brazil, especially those living in a situation of social vulnerability, such as the quilombola settlements in Brazil (2). Quilombola communities comprise ethnic-racial groups of black and African ancestry brought to Brazil and enslaved between the 16th and 19th centuries (3). After the abolition of slavery, communities emerged with their own sociocultural, economic, and religious characteristics that contributed to maintaining the particular ways of life within the quilombola communities and consolidating their territories (4, 5). There are 2,958 quilombola communities in Brazil certified by the Palmares Cultural Foundation, 35 of which are located in Sergipe (6–8). Despite globalization and modernization, these quilombola communities still have low socioeconomic and health conditions, with insufficient access to the healthcare system. Consequently, the quilombola communities are more vulnerable and susceptible to chronic diseases, such as systemic arterial hypertension (9–11), diabetes (12), and obesity (13), that, when associated with SARS-CoV-2, can result in a high mortality rate (9, 14).

The historic social vulnerability of quilombola communities has been a concern during the COVID-19 pandemic, a period also marked by the local political failure to implement fully adequate public protection policies (15, 16). For instance, quilombola communities still suffer from inadequate access to water and other basic infrastructure necessary for maintaining hygiene conditions, which are required to prevent the spread of the SARS-CoV-2 virus (17, 18). It is essential to mention that the World Health Organization (WHO) on 22 May 2020 declared Latin America as an epicenter of the coronavirus pandemic (19). Since there are several vulnerable areas in Latin America, it is necessary to sufficiently characterize the serological status and the circulation of SARS-CoV-2 in vulnerable communities of this local, as previously performed in Peru (20).

Notably, social vulnerability is primarily the case of rural areas of North and Northeast Brazil, where several quilombola communities live. In these regions, there was an inefficient implementation of the National Policy for Total Health for the Black Population (PNSIPN), which was created in 2009 (14, 21) inappropriate application of Law 14.021/21 that fundament the Emergency Plan to Combat COVID-19 for vulnerable communities, including Indians and Quilombolas (15, 17). The National Coordination for the Articulation of Black Rural Quilombola Communities in Brazil (CONAQ), in partnership with the Instituto Socio Ambiental, has launched a digital platform entitled “Observatório da COVID-19 nos Quilombos” to provide information on the number of COVID-19 cases (10). According to this platform, by January 12, 2022, there were 5,666 confirmed COVID-19 cases and 301 deaths in quilombola communitie (17). However, the serological status and the circulation of SARS-CoV-2 among quilombola communities and their relationship with the presence of risk factors or preexisting chronic diseases in the quilombola communities were not investigated so far (4–7). Thus, this study was performed to address this issue.

## Materials and methods

### Data collection in quilombola communities

**Figure 1:**
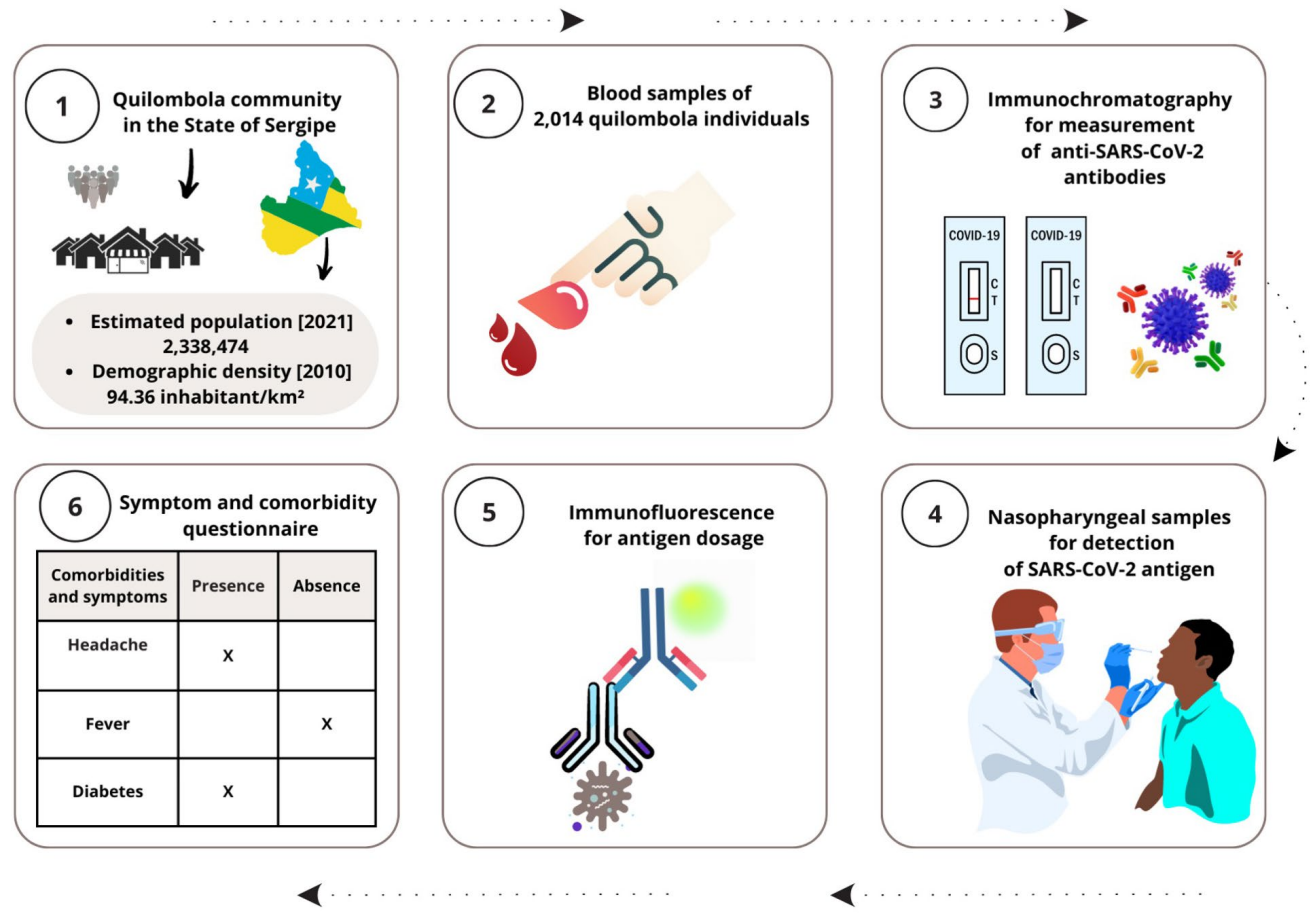
Study workflow. The images illustrate the steps of this work, showing local of data and sample acquisition as well as research approach. Created with Canva (http://www.canva.com)

Data were collected from 18 quilombola communities from 18 Sergipe municipalities. The workflow of this study is summarized in Figure 1. Each community was composed of an average of 100–150 people. We chose these communities because they are the most populous from 75 different municipalities of Sergipe, thus, covering the three Sergipe Mesoregions. One thousand nine hundred ninety-four individuals were included in our study. We used the following strategy to define the sample number in this study. Since there is an average of 150 individuals in each quilombola community, we assumed a sampling error of 5% and a confidence level of 95%, reaching 109 individuals as the ideal number for sampling. Samples were collected between August to October 2020, when vaccination was unavailable in Brazil; therefore, no individual in the quilombo community was vaccinated. The percentage of individuals with positive COVID-19 reactivity in the general population of the Sergipe state was obtained from the epidemiological Boletim (22).

The study was approved by the National Bioethics Committee of Brazil (CAAE 48254821.3.0000.5546). Participants were included in the study after providing informed consent.

### Sample selection

Participants were selected through random sampling, and we ensured that the sample was representative of the population under investigation (23). A simple random sample is one in which each member of the population has an equal chance of being selected. This sampling method is widely used in scientific research because it provides an unbiased population representation, allowing researchers to draw generalizable conclusions (24). Choosing a simple random sample begins with defining the population under investigation. Here, quilombola communities are registered with local city halls, and the number of members is limited and easy to access because they all live in the same place. To select the participants, we use a random number generator, a tool that generates a sequence of numbers that are random and unpredictable. In the case of simple random sampling, the numbers generated are used to select members of the population to be included in the sample (24).

### Demographic data

Demographic data of quilombola populations were obtained, such as age, sex, and socio-demographics. Medical records were analyzed using a validated questionnaire (25) from the Brazilian health surveillance for registration and notification of detectable cases of COVID-19 in Brazil through the e-SUS Notifica system.^1^ The questionnaire inquires whether the members of the quilombola communities had any comorbidities (e.g., hypertension, asthma, allergies, diabetes, and dyslipidemia, among others) and symptoms compatible with COVID-19, such as headache, runny nose, sneezing, cough, and fever.

### Laboratory analysis

To determine the COVID-19 positivity across the quilombola communities, we performed the Eco F COVID-19 Ag and the EGENS COVID-19 IgG/IgM Rapid Test. These two methods showed sensitivities of approximately 97% and specificity greater than 99.9%, as reported by the manufacturer. Both the lateral flow assay (EGENS COVID-19 IgG/IgM rapid test kit, Nantong Egens Biotechnology, Nantong, China) and the immunofluorescence assay (Eco F COVID-19 Ag kit with Eco Reader, Eco Diagnostica, Brazil) were previously reported (26–28). These tests have, in addition to the high sensitivity and specificity described above, the ease of execution in the quilombola region itself. The advantages of immunochromatography include its ability to be performed at the bedside without special laboratory equipment, its ease of performance, its simple interpretation, and it is rapid to produce results, which may compare favorably to RT-PCR (gold standard) and ELISA method (29). However, the sensitivity and specificity of immunochromatography greatly depend on the duration of infection and the antigens produced by manufacturers of the tests (30). It is important to note that at the time, in 2020, tests available and accessible in Brazil were scarce, and RT-PCR kits were unavailable due to high demand worldwide. Thus, the test that had the best performance and was available was chosen by the research group to carry out the study, being used in most of the other studies already published by the group (17, 19).

Nasopharyngeal samples were collected for detecting the SARS-CoV-2 antigen (reactivity: defined as reagent or non-reagent) using an immunofluorescence assay (Eco F COVID-19 Ag kit with Eco Reader, Eco Diagnostica, Brazil). Both rapid diagnostic tests applied in this study were performed in each quilombola community according to the manufacturer’s instructions.

IgM and IgG anti-SARS-CoV-2 antibodies (serological status: positive or negative) from serum samples were analyzed using lateral flow sandwich detection immunochromatography or finger-prick blood test (EGENS COVID-19 IgG/IgM rapid test kit, Nantong Egens Biotechnology, Nantong, China), validated by FDA (31) and described and another work of our group (32).

### Data analysis and visualization

Descriptive statistical and graphical analyses were performed using GraphPad Prism (version 8.0.0 for Windows, GraphPad Software, San Diego, California United States, www.graphpad.com) and rendered using Adobe Illustrator 2020 (Adobe, Ventura, CA, United States). Data from the federative unit of Sergipe were obtained through online access by the Secretary of Health of the State of Sergipe through the Observatory of the State of Sergipe (33). R software (version 4.1.1, R Foundation for Statistical Computing, Vienna, Austria; https://www.r-project.org/) was used to build a map of the geographic distribution of the reactive cases of IgG/IgM in quilombola communities in the municipalities of Sergipe (Brazil). We used the geobr package (34) to map the municipalities, which provides official spatial data sets of Brazil from the Brazilian Institute of Geography and Statistics (IBGE). We used the most recent dataset of the municipalities in Sergipe (2020) and created a map using the ggplot2 package (Springer-Verlang, New York, United States) (35).

We described categorical variables by absolute frequencies and percentages and continuous variables by median and interquartile range. We evaluated the independence hypothesis between categorical variables with Pearson’s Chi-Square test. We used the Poisson test to compare the prevalence of clinical manifestations. We observed a value of *p* under the adopted significance level of 5% in all cases mentioned in the manuscript.

We assessed the continuous variables normality adherence hypothesis with the Shapiro-Wilks test. Once this hypothesis was not met, we evaluated the hypothesis of equality of medians with the Mann–Whitney test. We also adopted a 5% significance level in all hypothesis tests.

## Results

### Quilombola communities present a higher percentage of SARS-COV-2 reactivity than the general population of Sergipe

**Figure 2:**
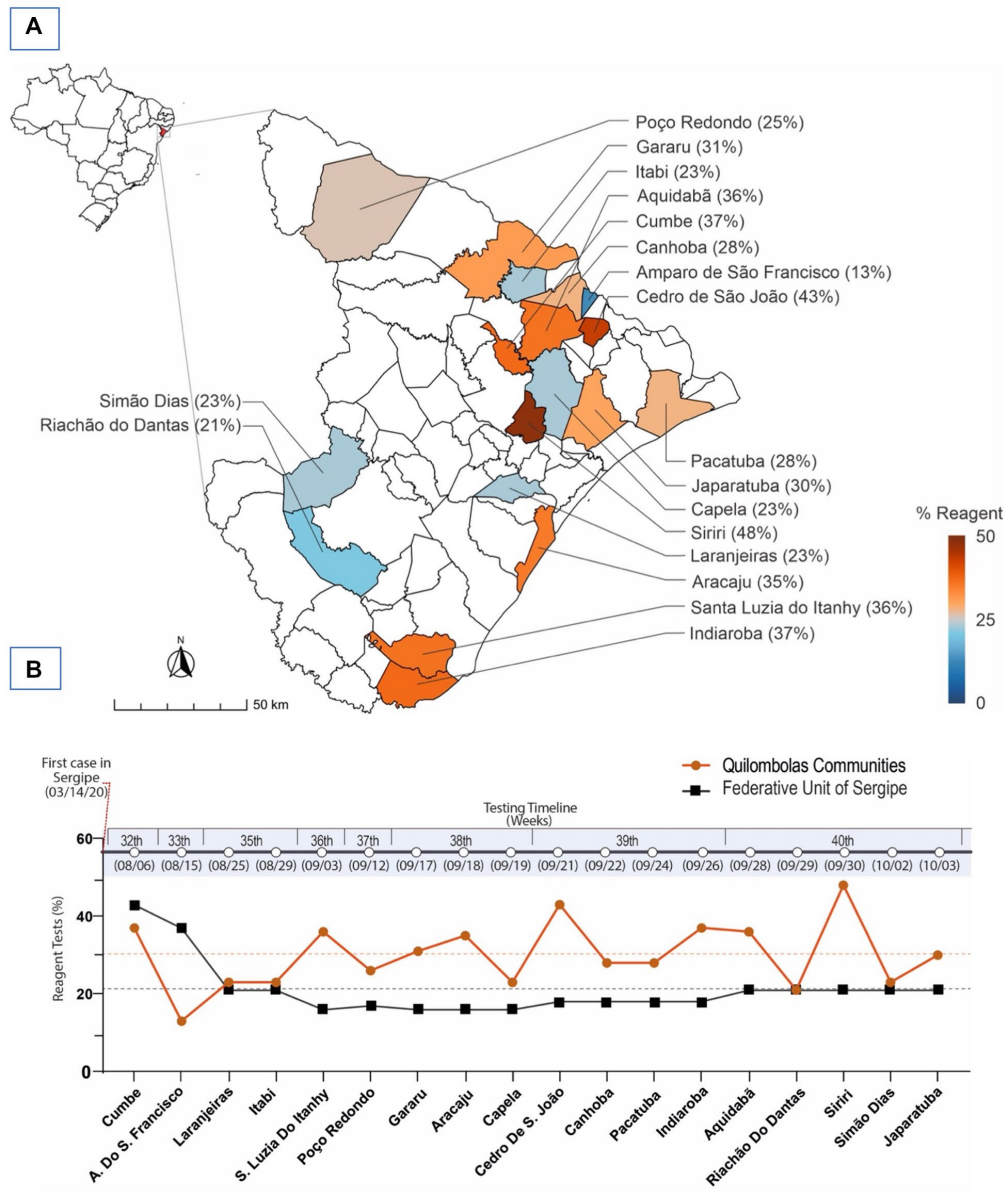
Geographical distribution of municipalities and quilombola communities in the state of Sergipe-Brazil and the detection of the SARS-CoV-2 antigen (reactivity). **(A)** The heat map represents the percentage of reactivity to the SARS-CoV-2 antigen, which was obtained from the nasopharyngeal samples across the quilombola communities in different municipalities. **(B)** The graphic shows the timeline of the reactivity of rapid tests for COVID-19, when the samples were collected in each quilombola community. It does not represent a longitudinal analysis, but the cross-sectional analysis (sample collection) of the 18 quilombola communities, which occurred at different time points. The *x*-axis shows the quilombola communities of the different municipalities tested (on the bottom) and the testing timeline in weeks (on the top). The *y*-axis indicates the % of reactivity. The orange and black lines represent the percentage reactivity (orange circles) of each quilombola community and the positivity of the average of the respective epidemiological week (black squares) in Sergipe.

We tested 1,994 individuals for COVID-19 reactivity (detection of the SARS-CoV-2 antigen) in 18 quilombola communities from Sergipe municipalities (Figure 2A). The quilombola communities sampled were distributed across different regions and were concentrated mainly in the north and east of the Sergipe state. Concerning the reactivity, nine quilombola communities had ≥30% of reagent cases, with the highest incidence in communities located in Siriri (48%of the Siriri quilombola community) and Cedro de São João (43% of the Cedro de São João quilombola community). Likewise, quilombola communities from nine municipalities had <30% of the reagent cases, with the lowest incidence in the community of Amparo de São Francisco (13%Amparo de São Francisco quilombola community). The mean positivity of the quilombola communities (from the 18 municipalities shown in Figure 2A) was 30.06%, i.e., higher than that observed in the general population of Sergipe (21.11%) (22).

Of note, the quilombola communities were evaluated at different epidemiological weeks, starting on the 32nd (August 6th) and ending on the 40th (October 3rd) week. Except for the two quilombola communities evaluated at the two first epidemiological weeks, all the others presented a higher % of reactivity when compared with the reactivity from the general population of Sergipe (Figure 2B).

### Serological, clinical evaluation, and sociodemographic of individuals of the quilombola communities and their association with SARS-CoV-2 infection

**Figure 3:**
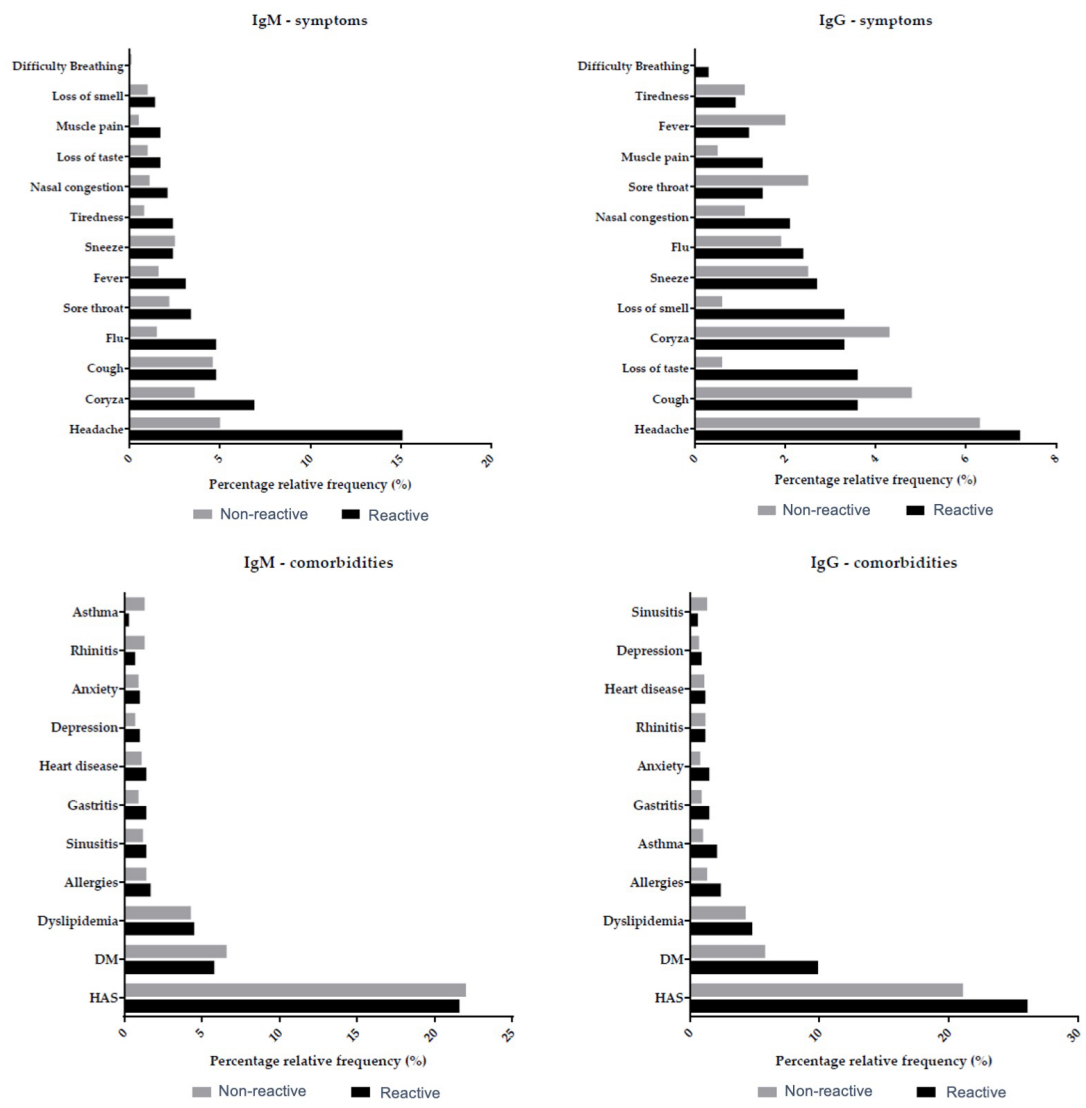
Serological data and clinical manifestations in the quilombola population. Association between IgM and IgG antibodies with symptoms and comorbidities verified in the quilombola population. Grey and black bars denote the percentage of non-reactive and reactive individuals in the quilombola population.

We next evaluated the serological status of the individuals from the quilombola communities and its relationship with their clinical manifestations. Of the total samples analyzed, 291 (14.6%) were positive for IgM and 333 (16.7%) for IgG. Meanwhile, 1,703 (85.4%) and 1,661 (83.3%) were negative for IgM and IgG, respectively (Table 1). In general, COVID-19-related symptoms were more frequent in individuals with IgM or IgG positive (reagent), such as muscle pain, headache, anosmia (loss of smell), and ageusia (loss of taste) (Figure 3 and Tables 2, 3).

**Table 1:** Serological status of the study cohort.

Serological status	*n*	%
IgM		
*positive*	291	14.6
*negative*	1703	85.4
IgG		
*positive*	333	16.7
*negative*	1,661	83.3

*n*, absolute frequency; %, percentage relative frequency.

**Table 2:** Demographic, laboratory, and clinical data in association with IgM reactivity.

	IgM		
	Reactive	Non-reactive	value of *p*
Age, Median (IIQ)	37 (27–48)	38 (27–51)	0.342^W^
Sex, *n* (%)			
*Male*	68 (23.4)	406 (23.8)	0.861^C^
*Female*	223 (76.6)	1,297 (76.2)	
SAP mmHg, Median (IIQ)	134.5 (120–150)	132 (120–150)	0.785^W^
DAP mmHg, Median (IIQ)	80 (70–90)	80 (70–90)	0.431^W^
AP classification, *n* (%)			
*Normal*	104 (35.7)	621 (36.5)	0.921^C^
*Bordeline normal*	47 (16.2)	258 (15.1)	
*Mild hypertension (stage 1)*	25 (8.6)	165 (9.7)	
*Moderate hypertension (stage 2)*	28 (9.6)	185 (10.9)	
*Severe hypertension (stage 3)*	25 (8.6)	126 (7.4)	
*Isolated systolic hypertension*	62 (21.3)	348 (20.4)	
Fasting Blood glucose mg/dL, Median (IIQ)	97 (91–108.75)	97 (91–107)	0.755^W^
Postprandial glycemia mg/dL, Median (IIQ)	105 (95–120)	109 (97–127)	**0.010** ^**W**^
Blood glucose mg/dL, Median (IIQ)	102 (93–118)	106 (95–122)	**0.039** ^**W**^
Blood glucose, *n* (%)			
> = 126	53 (18.2)	376 (22.1)	0.136^C^
<126	238 (81.8)	1,325 (77.9)	
RT-PCR, *n* (%)			
*Positive*	4 (33.3)	12 (18.5)	0.243^C^
*Negative*	8 (66.7)	53 (81.5)	
Allergies, *n* (%)	5 (1.7)	24 (1.4)	0.684^C^
Antigen, Median (IIQ)	0.05 (0.01–0.12)	0.08 (0.02–0.18)	**<0.001** ^**W**^
Anxiety, *n* (%)	3 (1)	16 (0.9)	0.882^C^
Asthma, *n* (%)	1 (0.3)	22 (1.3)	0.162^C^
Asymptomatic, *n* (%)	185 (63.6)	1,414 (83)	**<0.001** ^**C**^
Cardiopathy, *n* (%)	4 (1.4)	19 (1.1)	0.702^C^
Contact, *n* (%)	14 (4.8)	104 (6.2)	0.376^C^
Coryza, *n* (%)	20 (6.9)	62 (3.6)	**0.010** ^**C**^
Cough, *n* (%)	14 (4.8)	78 (4.6)	0.862^C^
Depression, *n* (%)	3 (1)	12 (0.7)	0.552^C^
Difficulty breathing, *n* (%)	0 (0)	1 (0.1)	0.679^C^
DM, *n* (%)	17 (5.8)	112 (6.6)	0.638^C^
Dyslipidemia, *n* (%)	13 (4.5)	74 (4.3)	0.925^C^
Fever, *n* (%)	9 (3.1)	28 (1.6)	0.091^C^
Flu, *n* (%)	14 (4.8)	26 (1.5)	**<0.001** ^**C**^
Gastritis, *n* (%)	4 (1.4)	16 (0.9)	0.491^C^
SAH, *n (%)*	**63 (21.6)**	**374 (22)**	**0.905** ^**C**^
Headache, *n* (%)	44 (15.1)	85 (5)	**<0.001** ^**C**^
Loss of smell, *n* (%)	4 (1.4)	17 (1)	0.561^C^
Loss of taste, *n* (%)	5 (1.7)	17 (1)	0.277^C^
Muscle pain, *n* (%)	5 (1.7)	8 (0.5)	**0.014** ^**C**^
Nasal congestion, *n* (%)	6 (2.1)	19 (1.1)	0.180^C^
No comorbidities, *n* (%)	188 (64.6)	1,048 (61.5)	0.319^C^
Rhinitis, *n* (%)	2 (0.7)	22 (1.3)	0.382^C^
Sinusitis, *n* (%)	4 (1.4)	20 (1.2)	0.772^C^
Sneeze, *n* (%)	7 (2.4)	43 (2.5)	0.904^C^
Sore throat, *n* (%)	10 (3.4)	37 (2.2)	0.189^C^
Tiredness, *n* (%)	7 (2.4)	14 (0.8)	**0.014** ^**C**^

*n*, absolute frequency; %, percentags; IIQ, interquartile range; SAP, systolic arterial pressure; DAP, diastolic arterial pressure; AP, arterial pressure; SAH: systemic arterial hypertension; W, Mann–Whitney test; C, Pearson’s Chi-square test; SD, standard deviation; DM, diabetes mellitus.

**Table 3:** Demographic, laboratory, and clinical data in association with IgG reactivity.

	IgG		
	Reactive	Non-reactive	value of *p*
Age, Median (IIQ)	38 (28–53)	38 (27–50.5)	0.563^W^
Sex, *n* (%)			
*Male*	66 (19.8)	408 (24.6)	0.063^C^
*Female*	267 (80.2)	1,253 (75.4)	
SAP mmHg, Median (IIQ*)*	137 (120–156)	132 (120–150)	0.111^W^
DAP mmHg, Median (IIQ)	80 (70–90)	80 (70–90)	0.211^W^
AP classification, *n* (%)			
*Normal*	111 (33.3)	614 (37)	0.315^C^
*Bordeline normal*	52 (15.6)	253 (15.2)	
*Mild hypertension (stage 1)*	29 (8.7)	161 (9.7)	
*Moderate hypertension (stage 2)*	47 (14.1)	166 (10)	
*Severe hypertension (stage 3)*	27 (8.1)	124 (7.5)	
*Isolated systolic hypertension*	67 (20.1)	343 (20.7)	
Fasting Blood glucose mg/dL, Median (IIQ)	99 (92–111)	97 (90–106)	0.135^W^
Postprandial glycemia mg/dL, Median (IIQ)	110 (97–128)	108 (97–126)	0.348^W^
Blood glucose mg/dL, Median (IIQ)	107 (95–124)	105 (95–121)	0.259^W^
Blood glucose, *n* (%)			
> = 126	79 (23.7)	350 (21.1)	0.287^C^
<126	254 (76.3)	1,309 (78.9)	
RT-PCR, *n* (%)			
*Positive*	10 (47.6)	6 (10.7)	**<0.001** ^C^
*Negative*	11 (52.4)	50 (89.3)	
Allergies, *n* (%)	8 (2.4)	21 (1.3)	0.113^C^
Antigen, Median (IIQ)	0.06 (0.01–0.13)	0.07 (0.01–0.16)	0.375^W^
Anxiety, *n* (%)	5 (1.5)	14 (0.8)	0.259^C^
Asthma, *n* (%)	7 (2.1)	16 (1)	0.076^C^
Asymptomatic, *n* (%)	264 (79.3)	1,335 (80.4)	0.648^C^
Cardiopathy, *n* (%)	4 (1.2)	19 (1.1)	0.929^C^
Contact, *n* (%)	28 (8.5)	90 (5.5)	**0.036** ^C^
Coryza, *n* (%)	11 (3.3)	71 (4.3)	0.415^C^
Cough, *n* (%)	12 (3.6)	80 (4.8)	0.336^C^
Depression, *n* (%)	3 (0.9)	12 (0.7)	0.731^C^
Difficulty breathing, *n* (%)	1 (0.3)	0 (0)	**0.025** ^C^
DM, *n* (%)	33 (9.9)	96 (5.8)	**0.005** ^C^
Dyslipidemia, *n* (%)	16 (4.8)	71 (4.3)	0.665^C^
Fever, *n* (%)	4 (1.2)	33 (2)	0.332^C^
Flu, *n* (%)	8 (2.4)	32 (1.9)	0.572^C^
Gastritis, *n* (%)	5 (1.5)	15 (0.9)	0.317^C^
HAS, *n* (%)	**87 (26.1)**	**350 (21.1)**	0.042^**C**^
Headache, *n* (%)	24 (7.2)	105 (6.3)	0.549^C^
Loss of smell, *n* (%)	11 (3.3)	10 (0.6)	**<0.001** ^C^
Loss of taste, *n* (%)	12 (3.6)	10 (0.6)	**<0.001** ^C^
Muscle pain, *n* (%)	5 (1.5)	8 (0.5)	**0.035** ^C^
Nasal congestion, *n* (%)	7 (2.1)	18 (1.1)	0.127^C^
No comorbidities, *n* (%)	193 (58)	1,043 (62.8)	0.097^C^
Rhinitis, *n* (%)	4 (1.2)	20 (1.2)	0.996^C^
Sinusitis, *n* (%)	2 (0.6)	22 (1.3)	0.269^C^
Sneeze, *n* (%)	9 (2.7)	41 (2.5)	0.803^C^
Sore throat, *n* (%)	5 (1.5)	42 (2.5)	0.259^C^
Tiredness, *n* (%)	3 (0.9)	18 (1.1)	0.766^C^

*n*, absolute frequency; %, percentages; IIQ, interquartile range; SAP, systolic arterial pressure; DAP, diastolic arterial pressure; AP, arterial pressure; SAH, systemic arterial hypertension; W, Mann–Whitney test; C, Pearson’s Chi-square test.

The clinical characterization of the quilombola populations showed a prevalence of arterial hypertension in 27.8% of the individuals (9.5% in stage 1, 10.8% in stage 2, and 7.5% in stage 3). Although post-glycemic glucose levels were not a concern, the mean fasting glucose values exceeded the limit of 100 mg/dL, indicating hyperglycemia (Table 4). The study population’s most common COVID-19 symptoms and comorbidities were headache, runny nose, flu, and dyslipidemia. However, most individuals were asymptomatic (79.9%) Table 5.

**Table 4:** Sociodemographic and clinical characteristics of the study cohort.

	*n*	%	Mean (SD)	Median (IIQ)
Age			39.49 (16.49)	38 (27–51)
Sex				
*Male*	478	23.7		
*Female*	1,536	76.3		
SAP mmHg			137.56 (25.32)	133 (120–150)
DAP mmHg			80.46 (14.83)	80 (70–90)
Normal pressure	733	36.4		
Borderline normal	309	15.3		
Mild hypertension (stage 1)	192	9.5		
Moderate hypertension (stage 2)	217	10.8		
Severe hypertension (stage 3)	152	7.5		
Isolated systolic hypertension	411	20.4		
Fasting blood glucose mg/dL			103.21 (27.55)	97 (91–107)
Postprandial blood glucose mg/dL			123.09 (55.01)	108 (97–126)
Blood glucose			118.38 (50.6)	105 (95–122)
> = 126	439	21.8		
<126	1,573	78.2		
SAH	442	21.9		
DM	133	6.6		

*n*, absolute frequency; %, percentage relative frequency; SD, standard deviation; IIQ, interquartile range; SAP; DAP; SAH, systemic arterial hypertension; DM, diabetes mellitus.

**Table 5:** Comorbidities and symptoms identified in the population tested.

	*n*	%
Dyslipidemia	88	4.4
Allergies	29	1.4
Asthma	23	1.1
Rhinitis	25	1.2
Sinusitis	24	1.2
Depression	16	0.8
Anxiety	19	0.9
Gastritis	21	1.0
Cardiopathy	23	1.1
Nasal congestion	26	1.3
Rhinitis	89	4.4
Headache	133	6.6
Fever	39	1.9
Cough	96	4.8
Sore throat	48	2.4
Loss of smell	21	1.0
Loss of taste	23	1.1
Tiredness	21	1.0
Sneeze	52	2.6
Difficulty breathing	1	0.05
Muscle pain	13	0.6
Flu	40	2.0
Asymptomatic	1,609	79.9
Contact	119	6
No comorbidities	1,245	61.8

*n*, absolute frequency; %, percentage/relative frequency.

Sociodemographic data revealed that the sample mainly comprised adults (mean age 39 years; SD16.49), males (23,7%), and females (76.3%) (Table 4). Among the quilombolas evaluated, 68 (23.4%) men and 223 (76.6%) women were positive for IgM; 26.8% had arterial hypertension classified in one of the three stages, and the prevalent symptoms were headache (15.1%), rhinitis (6.9%), flu (4.8%), cough (4.8%), and comorbid dyslipidemia (4.5%) (Table 2). Conversely, 66 men (19.8%) and 267 (80.2%) women had positive IgG; the most common symptoms in this group were headache (7.2%), cough (3.6%), loss of taste (3.6%) and runny nose (3.3%), as well as comorbid dyslipidemia (4.8%).

**Figure 4:**
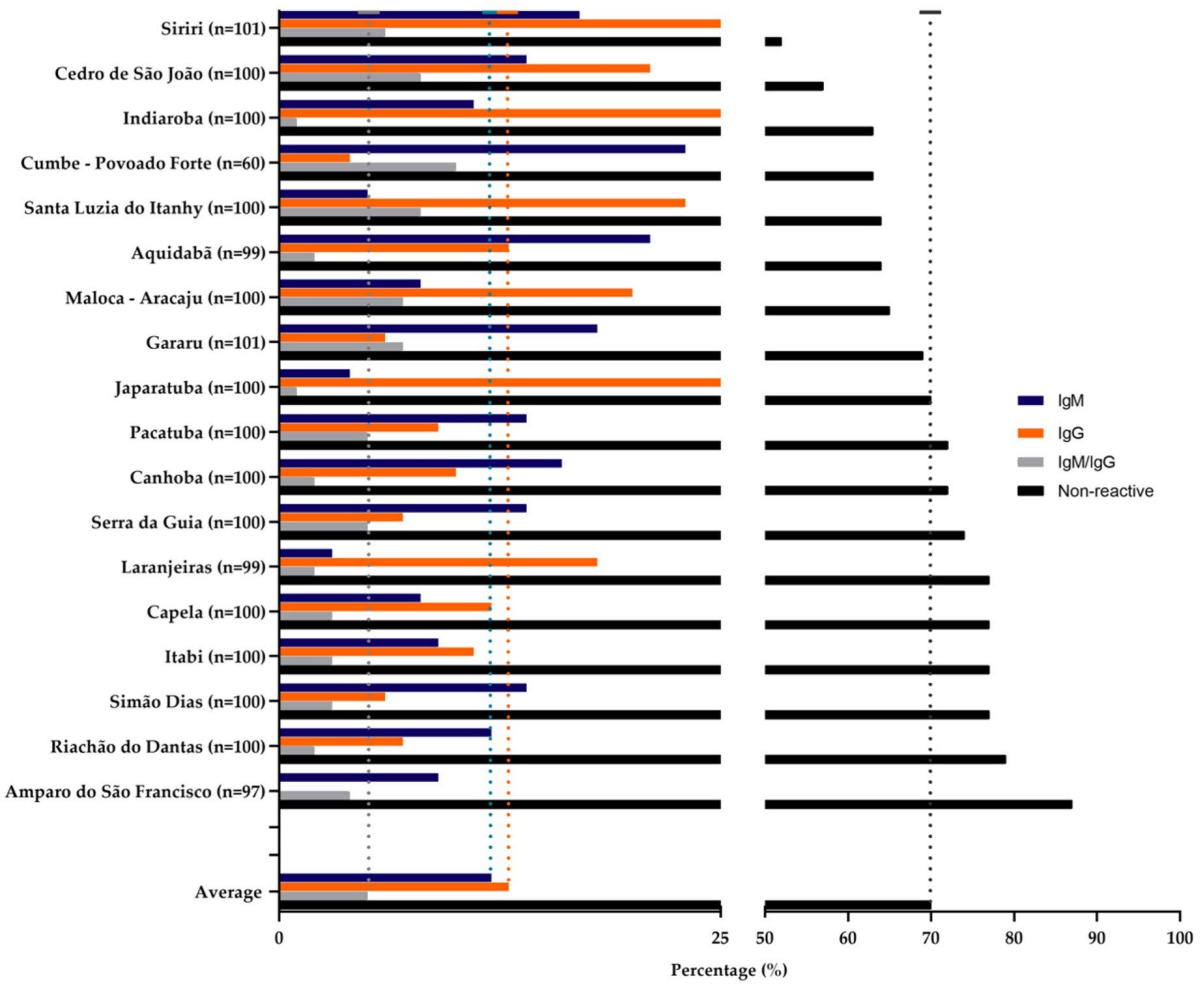
Quilombola communities and IgG/IgM reactivity. Black, orange, and grey bars represent the percentage of non-reactive and reactive individuals in the quilombola population for each municipality.

Regarding the communities, Povoado Forte, in the municipality of Cumbe, was the one that presented the most individuals with IgM reagent, followed by the communities present in Aquidabã and Gararu (Figure 4). The communities in Siriri, Indiaroba, and Japaratuba had more IgG-positive individuals. Amparo of São Francisco was a municipality with higher non-reactive values for the antibodies tested. A greater tendency towards seropositivity was observed in the Povoado Forte community, followed by those in Cedro de São João and Santa Luzia do Itanhi.

## Discussion

This cross-sectional study characterizes the SARS-COV-2 infection profile in quilombola communities in Brazil, which are under social vulnerability, representing an issue of concern highly associated with high infection rates during the COVID-19 pandemic (8). In agreement, we found that there is a higher percentage of SARS-COV-2 reactivity in quilombola communities than in the population of Sergipe. Typical COVID-19-related symptoms (e.g., muscle pain, headache, anosmia, and ageusia (loss of taste) were frequently demonstrated by IgM- or IgG-positive (reagent) individuals. Furthermore, most individuals affected by COVID-19 were female, which is in agreement with previous findings by Borges et al. (17) and Gonçalves et al. (8). However, we cannot conclude that this result means that females are more affected than males in these populations because, in addition to the selection bias in the study participants (>3 females/1 male), Sergipe is a state with a higher number of females (about 51.74%) to male population (with 48,26%) (36). To the best of our knowledge, this work characterizes for the first time the association between sociodemographic or risk factors and chronic diseases with the SARS-CoV-2 infection profile (symptoms and comorbidities) of individuals from the quilombola communities.

This study highlights the importance of precise COVID-19 diagnosis, neglected in quilombola communities, despite being essential for individuals’ health and social care to delay SARS-CoV-2 transmission. This is highly important since there was a higher incidence of SARS-CoV-2 infection in the quilombola communities compared to the general Sergipe population. In this context, specific IgM antibodies for SARS-CoV-2 can be detected approximately 3–5 days after the onset of infection, and 14.6% of the individuals from the quilombola communities were IgM-positive. Nonetheless, although they were acutely infected, they lived in free contact with the other 85.4% of uninfected individuals in these communities. Furthermore, the indices of IgG-positive individuals indicated that 16.7% of the quilombola populations were with late, secondary, or previous SARS-CoV-2 infection. Despite that, the individuals who participated in our study were followed up by municipal health departments. No adverse outcomes (disease worsening, resulting hospitalization or death) were reported 12 months after testing.

Although a systematic review and meta-analysis of the SARS-CoV-2 seroprevalence worldwide revealed that the SARS-CoV-2 seroprevalence varied markedly among geographic regions (37), our data is following the epidemiological studies in Africa showing a lower incidence of severe COVID-19 than in developed countries (38). However, although there are different hypotheses to explain these better outcomes in Africa compared to other continents, it remains undetermined. Therefore, future studies are essential to investigate the relationship between the hygiene hypothesis, the COVID-19 pandemic, and the human microbiome in the epidemiology of SARS-CoV-2 infections (39). These better outcomes in low-income areas are even more surprising because comorbidities representing risk factors for severe COVID-19, such as arterial hypertension and diabetes (40), were highly presented in our study cohort and frequently reported in quilombola communities (9). Of note, a 26% prevalence of arterial hypertension is reported in the quilombola communities of Sergipe, which is higher than the standard observed in the general population (20.4%) in the same state (9). Likewise, we also observed the incidence of diabetes (DM) in the population tested, as reported by Roriz et al. (41) in patients from a hospital in the state of Sergipe, and the indication of hyperglycemia, which may be related to the risk of developing metabolic syndrome and has already been observed predominantly in quilombola women (42). Despite that, most IgM and IgG seropositive cases were asymptomatic (79.9%), as Borges et al. (43) observed in the general population.

Among symptomatic patients, headache was the most common complaint in our study, in accordance with a previous report showing that headache is a symptom often associated with COVID-19, requiring clinical attention because it can persist beyond the acute disease phase as a persistent symptom (44). Furthermore, we also observed the presence of cough in seropositive individuals (4.8%), as found by Adil et al. (45) and Yang et al. (46), with cough being the second most frequent symptom reported by these authors. In addition, some studies considered coughing to be a trigger for headaches, which may explain the higher incidence of both symptoms in the population tested (44). Although we found rhinitis o be the third most frequent symptom (4.4%), there are few reports of patients with this symptom in the literature (47, 48). We also found the loss of smell and taste frequently reported in our cohort, which are hallmark symptoms of COVID-19 (49). We saw similar symptoms and comorbidities in the SARS-CoV-2 positive and negative groups. This fact might be explained by the cocirculation of SARS-CoV-2 and other respiratory viruses, such as influenza viruses, respiratory syncytial virus, or adenoviruses, enhancing the respiratory disease burden worldwide and causing overlapping symptoms in SARS-CoV-2 positive and negative individuals (50, 51).

Despite being relevant to characterize the clinical profile and the high number of SARS-COV-2 infections in quilombola communities, this study has limitations. We could have used more accurate tests (e.g., RT-PCR) to evaluate our study cohort’s reactivity and serological status. However, the anti-SARS-CoV-2 IgM and IgG antibodies were tested using an approved approach by the Brazilian Health Regulatory Agency (ANVISA; Registration number: 814647500072) and approved by the FDA (31). This test has been used frequently in our studies (26) and qualitatively detects IgM and IgG antibodies separately with a sensitivity of 96.8% and specificity of 96.52% (52, 53). Another limitation of our study is that we performed a cross-sectional study. Thus, we do not follow the epidemiological dynamics of SARS-CoV-2 infections during the COVID-19 pandemic in the quilombola communities. Another limitation is that despite not representing a comprehensive epidemiological analysis, this study is an investigation of social importance in under-tested people, acting as an instrument for understanding the pandemic in these communities.

In conclusion, there was a higher number of SARS-COV-2 infections in quilombola communities than in the local population in Brazil when we developed this study. Testing for the SARS-CoV-2 infectious status could have played a significant role in the transmission of this virus across the quilombola populations, particularly during the pandemic when vaccines were unavailable. However, this population presented favorable disease outcomes. Thus, political initiatives developed by the current study should be supported by local governments and broadly implemented through public policy to mitigate the damage to quilombola populations during future pandemics or epidemic events.

## Data availability statement

The original contributions presented in the study are included in the article/Supplementary material, further inquiries can be directed to the corresponding authors.

## Ethics statement

The studies involving human participants were reviewed and approved by Ethics Committee of the Federal University of Sergipe (CAAE 48254821.3.0000.554 and date 01/11/2021). The patients/participants provided their written informed consent to participate in this study.

## Author contributions

AM, LB, DS, JR, AS, GI, IM, KS, PJ, FS, FA, SM, BO, IC, and OM: conceptualization and methodology. FV, DF, LS, SS, RG, and BP: figures and tables. CF, BP, CB, SS, RG, LB, BM, and OM: writing—preparation of the original draft. BP, CF, AM, LB, CB, FV, DF, LS, OM, and BM: writing—proofreading and editing. All authors contributed to the article and approved the submitted version.

## Funding

This work was supported by CAPES (finance code 001) to FV; the National Council for Scientific and Technological Development (CNPq), Brazil (grants: 309482/2022-4 to OM and 102430/2022-5 to LS); Federal University of Ouro Preto (grant number 23109.000928/2020-33); São Paulo Research Foundation (FAPESP grant 2018/18886-9 to OM and 2020/16246-2 to DF).

## Conflict of interest

The authors declare that the research was conducted in the absence of any commercial or financial relationships that could be construed as a potential conflict of interest.

## Publisher’s note

All claims expressed in this article are solely those of the authors and do not necessarily represent those of their affiliated organizations, or those of the publisher, the editors and the reviewers. Any product that may be evaluated in this article, or claim that may be made by its manufacturer, is not guaranteed or endorsed by the publisher.

## Footnotes

^1^ https://notifica.saude.gov.br/login
